# Curcumin and inflammation in non-alcoholic fatty liver disease: a randomized, placebo controlled clinical trial

**DOI:** 10.1186/s12876-019-1055-4

**Published:** 2019-07-25

**Authors:** Saeede Saadati, Amir Sadeghi, Asieh Mansour, Zahra Yari, Hossein Poustchi, Mehdi Hedayati, Behzad Hatami, Azita Hekmatdoost

**Affiliations:** 1 Department of Clinical Nutrition and Dietetics, Faculty of Nutrition and Food Technology, National Nutrition and Food Technology, Research Institute, Shahid Beheshti University of Medical Sciences, Tehran, Iran; 2 Gastroenterology and Liver Diseases Research Center, Research Institute for Gastroenterology and Liver Diseases, Shahid Beheshti University of Medical Sciences, Tehran, Iran; 3 Liver and pancreatobiliary research group, Digestive Disease Research Institute, Tehran, Iran; 4 Cellular and Molecular Endocrine Research Center, Research Institute for Endocrine Sciences, Shahid Beheshti University of Medical Sciences, Tehran, Iran

**Keywords:** NAFLD, Fatty liver, Curcumin, Inflammation, NASH

## Abstract

**Background:**

The aim of the present study was to evaluate the effects of curcumin supplementation on inflammatory indices, and hepatic features in patients with non-alcoholic fatty liver disease (NAFLD).

**Methods:**

Fifty patients with NAFLD were randomized to receive lifestyle modification advice plus either 1500 mg curcumin or the same amount of placebo for 12 weeks.

**Results:**

Curcumin supplementation was associated with significant decrease in hepatic fibrosis (*p* < 0.001), and nuclear factor-kappa B activity (*p* < 0.05) as compared with the baseline. Hepatic steatosis and serum level of liver enzymes, and tumor necrosis-α (TNF-α) significantly reduced in both groups (*p* < 0.05). None of the changes were significantly different between two groups.

**Conclusion:**

Our results indicated that curcumin supplementation plus lifestyle modification is not superior to lifestyle modification alone in amelioration of inflammation.

**Trial registration:**

IRCT20100524004010N24, this trial was retrospectively registered on May 14, 2018.

## Background

As a major health problem, NAFLD (Non-alcoholic Fatty Liver Disease) has been attracted more attention over the past few years [1, 2], which has more harmonious relation with high prevalence of metabolic ailments including obesity and diabetes [2]. On the other hand, high prevalence (75%) of NAFLD is observed in obese individuals and even more in type 2 diabetes mellitus patients, while this number is as high as 17–35% in the general population [3, 4].

NAFLD is characterized by triacylglycerol build-up (> 5–10% of hepatocytes) in the absence of significant alcohol consumption in early stages, which can progress to the more crucial non-alcoholic steatohepatitis (NASH) and in the end stages to the hepatic cirrhosis and hepatocellular carcinoma [5].

With respect to the multiple hit hypothesis, mitochondrial dysfunction and oxidative stress appear to be nearly the most important mechanisms, regardless of initial causes in the pathophysiology of NAFLD/NASH [6, 7]. Accumulation of lipids in the hepatocytes causes redox imbalance and triggers fat oxidation. Hence, inflammatory response along with up-regulation of pro-inflammatory cytokines including tumor necrosis factor α (TNF-α), and high-sensitivity C-reactive protein (hs-CRP) come about [5, 6]. Cornerstone of NAFLD management is lifestyle modification [8], although combination of it with anti-inflammatory agents improves the treatment in the best way [9–14].

A major and active constituent of turmeric is a yellow pigment isolated from *Curcuma longa Linn,* which is named as curcumin [15]. Recent in vitro and in vivo studies have shown the anti-oxidant and anti-inflammatory properties of curcumin [16, 17]. Curcumin allayed the severity of hepatic inflammation in experimental model of steatohepatitis [18]. Curcumin was reported to be useful in the modulation of oxidative stress condition and inflammation cascades in rats on high fructose diets by regulating the expression of nuclear factor- kappa B (NF-κB) in hepatocytes [19]. On the other hand, Rahmani et al. have shown the beneficial effects of curcumin on metabolic features of NAFLD [20]; however, its role in management of inflammation in NAFLD has not yet been elucidated in human [21, 22]. Hence, the objective of the present study was to evaluate the effects of curcumin supplementation on inflammatory indices, and hepatic features in patients with NAFLD.

## Methods

### Subjects

This study was a placebo-controlled, double-blinded, randomized clinical trial, which was designed according to CONSORT guideline. Participants were recruited from gastroenterology and liver clinics of Taleghani hospital (A tertiary center in Tehran, Iran). The study population was comprised of subjects who were 18 years or older with evidence of hepatic steatosis using Fibroscan (Echosens, Paris, France) (Controlled Attenuation Parameter (CAP) > 263 dB/m) and who exhibited the following inclusion criteria:

No history of alcohol consumption or consumption of less than 20 g of alcohol per day in women and less than 30 g per day in men; absence of other liver disorders, malignancies, cardiovascular, respiratory and kidney disorders, no history of weight loss or bariatric surgery in recent years, absence of medication consumption in the previous 3 months, and absence of endocrine and metabolic disorders. Exclusion criteria were pregnancy or breastfeeding, lack of compliance with the supplementation (defined as who had not consumed 90% of expected capsules on last visit), participation in a concomitant trial; hypersensitivity to the supplementation; and preference for not continuing the study. Informed consent was obtained from each patient who was included in the trial. The study protocol was approved by the Ethics Committee of National Nutrition and Food Technology Research Institute at Shahid Beheshti University of Medical Sciences (IR.SBMU.nnftri.Rec.1395.106). The study protocol was registered at clinicaltrial.gov with registration number of NCT02908152.

### Study design

The sample size was calculated according to the serum TNF-α. Calculation of the sample size for this study was based on detection of a 2 unit (pg/mL) difference in the mean TNF-α score with a power of 80% (β = 20%), yielding a sample size of 21 for each group. Given the possible potential loss of samples, 25 patients in each group were considered [3]. Eligible participants who met the inclusion criteria were randomly assigned to receive either 500 mg curcumin or matched placebo TID (3 times a day) after each meal for 12 weeks. Randomization lists were computer-based by a statistician and the participants and project managers were completely unaware (blind) about the intervention and control groups. BCM-95(BIO-CURCUMIN®) is a proprietary combination of 95% curcuminoids, ensuring a high level of bioavailability of curcumin. It does not contain non-turmeric components anymore to induce curcumin more bioactivity. Placebo capsules were identical capsules containing the same amount of maltodextrin. Both curcumin and placebo (Maltodexterin) capsules were produced by Arjuna Natural Extract, India. The concealments of supplements were done by a third person, and the participants and investigators were not aware of the groups’ assignments until the end of analysis. At the beginning of the study, the implementation of the study was discussed with the participants and the supplements were delivered for as much as 3 weeks utilities. Follow-up visits were carried out every 3 weeks while the supplements were delivered and nutrition counseling was done.

Both groups were advised to undergo an energy-balanced diet and follow physical activity recommendations according to the clinical guidelines from the national institutes for health (NIH) and the North American Association for the Study of Obesity [23]. Nutrient distribution was as follow: 52 to 55% of energy from carbohydrates, less than 30% of energy from lipids and 15–18% of energy from proteins were provided. As well as, participants were advised to limit their dietary cholesterol intake to less than 300 mg per day and consume 20 to 30 g of fiber per day. Furthermore, they were advised to exercise ≥30 min, three times per week. Phone calls were made to remind the participants about supplement consumption at the end of each week.

### Clinical, paraclinical, and dietary intake assessments

Anthropometric indices including weight, height, and waist were measured for all participants at the baseline and the end of study. Measurement of weight with 100 g accuracy using digital scale for participants wearing minimal clothing and no shoes and height with 0.5 cm accuracy using a tape measure while the participants were standing in a normal position with no shoes was carried out. Body mass index (BMI) was calculated as weight (in kilograms) divided by the square of height (in meters). To avoid measurement error, all measurements were accomplished by the same person. After 12 h fasting, with respect to standard protocol, blood samples were collected at the beginning and end of week 12. All biochemical tests were precisely assessed in the same laboratory. Serum concentration of alanine aminotransferase (ALT), and aspartate aminotransferase (AST) were assessed using enzymatic methods (Parsazmun Co, Tehran, Iran). The concentration of TNF-α was measured using an enzyme-linked immunosorbent assay (ELISA) kit (eBioscience, Inc., San Diego, CA, USA, Cat No: 109957025) and high-sensitivity C-reactive protein (hs-CRP) concentration was also measured by ELISA kit (Parsazmun Co., Tehran, Iran, Cat No: 93001). By using ELISA kit (Cell signaling, Danvers, MA, USA). NF-κB p65 was measured in peripheral blood mononuclear cells (PBMCs) nuclear extracts, according to the manufacture’s protocol.

At the entry and the end of study, hepatic fibrosis and steatosis were evaluated using Fibroscan® (Echosens, Paris, France) [24]. This assay was performed by the same operator who was blinded to the study randomization. Moreover, physical activity of participants was evaluated using the metabolic equivalent of task (MET) questionnaire [25]. For dietary intake assessment, all of the participants were instructed how to fill in the daily dietary record for 3 days (one weekend day and two workdays) at baseline and end of the study. Afterward, dietary intakes were analyzed using the US Department of Agriculture and National Food Composition tables [26].

### Primary and secondary outcomes

The primary outcome was a significant reduction in TNF-α concentration in curcumin grou*p* compared to placebo group. Secondary outcome measures were serum hs-CRP, NF-kB activity in PBMCs, and hepatic steatosis and fibrosis.

### Statistical analyses

Analysis of collected data was done using SPSS version 21. The normality of distribution of variables was assessed by Kolmogorov-Smirnov and Shapiro-Wilk tests. Statistically significance was presented as *p* value < 0.05. Demographic data analysis was done using either t test or chi-square test. If distribution of data was normal, paired t test and student t test were used to compare variables within and between groups, respectively. But if not, Wilcoxon and Mann-Whitney U tests substitute. In favor of annihilation of confounding factors effects, in the baseline and/or during the study, the analysis of covariance test was utilized. *P* value < 0.05 was considered as significant.

## Results

**Fig. 1:**
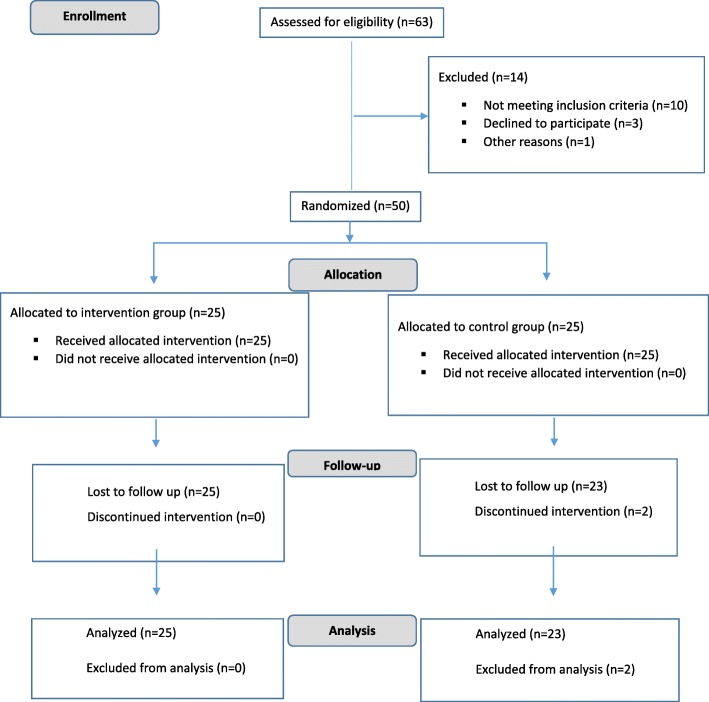
Consort flow chart of the study

Fifty patients were enrolled in this study. Only two patients (8%) were dropped out due to being not interested in continuing the study (Fig. 1). Totally 48 patients completed the study in specified period of study. Curcumin was safe and well tolerated and none of participants reported any adverse effects. Baseline values of demographic and metabolic factors are shown in Table 1. There was not any significant difference between two groups in terms of age, sex, anthropometric indices, daily energy intake and physical activity level at baseline (Table 1). No significant difference was detected between two groups in serum level of TNF-α, hs-CRP and NF-κB, and hepatic characteristics (Table 1).

**Table 1:** Baseline characteristics of patients with NAFLD participated in our study before intervention^a^

Variables	Placebo group	Curcumin group	*P* value
Age (year)	10.9 ± 45.13	11.5 ± 46.19	0.473
Sex (male, n,(%))	14 (51.9%)	13 (48.1%)	0.407
Physical Activity (MET.h.d)	4.63 ± 32.03	3.65 ± 32.69	0.592
Smoking			0.167
Yes (Smoker or Ex-smoker)	4 (17%)	1 (4%)	
No (Never smoked)	19 (83%)	26 (96%)	
Weight (kilogram)	89.22 ± 13.05	85.02 ± 11.16	0.225
BMI (kg/m^2^)	32.38 ± 5.02	32.30 ± 4.55	0.956
Waist circumference (cm)	103.28 ± 8.83	102.19 ± 8.78	0.948
Total energy (kcal)	2323.01 ± 540.59	2355.41 ± 703.69	0.858
hs-CRP (ng/dL)	6705.05 ± 4797.9	5647.15 ± 3858.4	0.412
TNF-α (pg/mL)	18.63 ± 2.2	19.34 ± 5.4	0.610
NF-κB	2.13 ± 0.92	2.07 ± 1.03	0.842
Fibrosis grade (kPa)	6.52 ± 2.38	6.98 ± 2.42	0.132
Steatosis grade (db/m)	315.18 ± 35.69	298.35 ± 29.5	0.218

^a^Data are presented as mean ± SD except for gender, which is reported as number (%)

*NAFLD* none-alcoholic Fatty Liver Disease, *BMI* body mass index, *TNF* tumor necrosis factor, *NF-kB* Nuclear factor kappa B, *hs-CRP* high sensitive C reactive protein

Within both groups, significant decrease in the weight, BMI, and waist circumference have been demonstrated; however, no significant difference was detected between two groups (*p* > 0.05). Within both groups, daily energy intake decreased significantly while there was no significant difference between changes in two groups. In curcumin group, physical activity increased significantly more than placebo group.

Curcumin supplementation was associated with significant decrease in hepatic fibrosis (*p* < 0.001) and the activity of NF-κB in PBMCs (*p* < 0.05) as compared with the baseline (Table 2). Within both groups, serum level of hs-CRP reduced but this reduction was not significant (*p* > 0.05) (Table 2). Hepatic steatosis and serum level of ALT, AST, and TNF-α reduced significantly in both groups after 12 weeks of the study intervention (Table 2). None of the changes during study intervention were significantly different between two groups (Table 2).

**Table 2:** Inflammatory biomarkers and hepatic characteristic changes after 12 week of intervention

characteristic	Baseline	After 12 week	*P* ^a^	Change (%)	*P* ^b^
hs-CRP (ng/dL)					
Curcumin group	5647.1 ± 3858.4	4653.8 ± 4243.8	0.12	− 993.3 ± 385.4	0.660
Placebo group	6705.0 ± 4794.9	5310.2 ± 5071.7	0.53	− 1394.8 ± 276.8	
TNF-α (pg/mL)					
Curcumin group	19.34 ± 5.4	16.51 ± 4.5	< 0.001	−2.83 ± 0.9	0.972
Placebo group	18.63 ± 2.2	16.54 ± 2.1	0.02	−2.09 ± 0.1	
NF-κB					
Curcumin group	2.07 ± 1.03	1.65 ± 0.63	0.044	−0.49 ± 0.29	0.539
Placebo group	2.13 ± 0.92	2.03 ± 0.51	0.209	− 0.26 ± 0.20	
ALT (IU/L)					
Curcumin group	26.54 ± 15.46	20.82 ± 10.09	< 0.001	−5.58 ± 14.6	0.778
Placebo group	28.02 ± 13.06	21.2 ± 7.72	< 0.001	−6.82 ± 15.8	
AST (IU/L)					
Curcumin group	17.78 ± 9.56	14.75 ± 7.45	< 0.001	−2.76 ± 7.54	0.728
Placebo group	16.23 ± 5.66	12.77 ± 4.05	< 0.001	−3.46 ± 5.43	
Fibrosis (kPa)					
Curcumin group	6.98 ± 2.42	6.2 ± 2.38	< 0.001	−0.78 ± 0.89	0.364
Placebo group	6.52 ± 2.38	6.02 ± 1.8	0.095	−0.49 ± 1.15	
Steatosis (db/m)					
Curcumin group	298.35 ± 29.5	282.65 ± 40.09	0.015	−15.69 ± 30.72	0.112
Placebo group	315.18 ± 35.69	283.18 ± 49.83	0.001	−32 ± 34.3	

*NAFLD* none-alcoholic Fatty Liver Disease, *BMI*: body mass index, *TNF* tumor necrosis factor, *NF-kB* Nuclear factor kappa B, *hs-CRP* high sensitive C reactive protein, *ALT* Alanine aminotransferase, *AST* Aspartate aminotransferase

^a^*P* values indicate comparison within groups

^b^*P* values indicate comparison between the changes of each variable between 2 groups

## Discussion

Our results have shown that supplementation with 1.5 g/d curcumin besides lifestyle modification for 12 weeks is not superior to lifestyle modification alone in amelioration of inflammation in patients with NAFLD. Previous preclinical studies have shown that curcumin modified oxidative stress condition and inflammation cascades through controlling the serum level of glucose, insulin, leptin, cholesterol, triglycerides and expression of NF-κB in hepatocytes of rats on high fructose diets [27, 28]. Furthermore, curcumin subsided the expression of TNF-α, CRP, cyclooxygenase 2 and protein kinase C and inhibited the activation of NF-κB in high fructose fed male Wistar rats [29]. Another study suggested that curcumin can ameliorate rabbit’s steatohepatitis induced by high fat diet through diminishing TNF-α production [30].

One of the key mechanisms that have been attributed to anti-inflammatory effects of curcumin is the suppression of NF-κB [31]. Our study is the first human study that evaluated the effects of curcumin supplementation on NF-κB expression in PBMCs of NAFLD patients. Our results have shown that curcumin significantly reduced NF-kB expression; however, this reduction was not significantly different from placebo group. This discrepancy can be due to significant weight reduction in both groups, which is the only proven strategy in management of NAFLD (8). Weight reduction inhibits accumulation of lipid in hepatocytes, resulting in reduction of inflammatory response via NF-κB activation and cytokine production, which leads to decrease in insulin resistance [32].

This study has some strengths; it was the first clinical trial that evaluated the effects of curcumin on inflammatory markers in NAFLD patients; using transient elastography for hepatic steatosis and fibrosis assessment was another advantage of this study; placebo-controlled design of the study provided us the advantage of assessment of superiority of curcumin supplementation plus lifestyle modification to lifestyle modification alone.

## Conclusion

In conclusion, our results indicated that curcumin supplementation plus lifestyle modification is not superior to lifestyle modification alone in amelioration of inflammation and hepatic steatosis and fibrosis.

## Data Availability

The datasets generated during the current study are available from the corresponding author on reasonable request.

## Abbreviations

BMI: Body mass index
ELISA: Enzyme-linked immunosorbent assay
hs-CRP: High sensitive C reactive protein
NAFLD: None-alcoholic fatty liver disease
NASH: None-alcoholic steatohepatitis
NF-kB: Nuclear factor kappa B
TNF: Tumor necrosis factor

## References

[1] Mokhtari Z, Gibson DL, Hekmatdoost A (2017). Nonalcoholic fatty liver disease, the gut microbiome, and diet. Adv Nutr.

[2] Younossi ZM (2019). Non-alcoholic fatty liver disease—a global public health perspective. J Hepatol.

[3] Eslamparast T (2014). Synbiotic supplementation in nonalcoholic fatty liver disease: a randomized, double-blind, placebo-controlled pilot study. Am J Clin Nutr.

[4] Foschi FG (2018). Prevalence of and risk factors for fatty liver in the general population of northern Italy: the Bagnacavallo study. BMC Gastroenterol.

[5] Byrne CD, Targher G (2015). NAFLD: a multisystem disease. J Hepatol.

[6] Buzzetti E, Pinzani M, Tsochatzis EA (2016). The multiple-hit pathogenesis of non-alcoholic fatty liver disease (NAFLD). Metabolism.

[7] Darand M, Alavian SM, Hekmatdoost A (2018). Nigella sativa and non-alcoholic fatty liver disease: a review of the current evidence. Hepat Mon.

[8] Ghaemi A, et al. How much weight loss is effective on nonalcoholic fatty liver disease? Hepat Mon. 2013;13(12). 10.5812/hepatmon.15227 PMC3867211 24358045

[9] Emamat H (2015). The effects of onion consumption on treatment of metabolic, histologic, and inflammatory features of nonalcoholic fatty liver disease. J Diabetes Metab Disord.

[10] Hekmatdoost A (2016). Adherence to the dietary approaches to stop hypertension (DASH) and risk of nonalcoholic fatty liver disease. Int J Food Sci Nutr.

[11] Mokhtari Z (2017). Egg consumption and risk of non-alcoholic fatty liver disease. World J Hepatol.

[12] Noori M, Jafari B, Hekmatdoost A (2017). Pomegranate juice prevents development of non-alcoholic fatty liver disease in rats by attenuating oxidative stress and inflammation. J Sci Food Agric.

[13] Rahimlou M, Ahmadnia H, Hekmatdoost A (2015). Dietary supplements and pediatric non-alcoholic fatty liver disease: present and the future. World J Hepatol.

[14] Rahimlou M (2016). Ginger supplementation in nonalcoholic fatty liver disease: a randomized, double-blind, placebo-controlled pilot study. Hepat Mon.

[15] Panahi Y (2016). Mitigation of systemic oxidative stress by Curcuminoids in osteoarthritis: results of a randomized controlled trial. J Diet Suppl.

[16] Alm-Eldeen AA (2015). Synergistic effect of black tea and curcumin in improving the hepatotoxicity induced by aflatoxin B1 in rats. Toxicol Ind Health.

[17] Dattani J (2010). Ameliorative effect of curcumin on hepatotoxicity induced by chloroquine phosphate. Environ Toxicol Pharmacol.

[18] Coban D (2012). Dietary curcumin inhibits atherosclerosis by affecting the expression of genes involved in leukocyte adhesion and transendothelial migration. Mol Nutr Food Res.

[19] Leclercq IA (2004). Curcumin inhibits NF-κB activation and reduces the severity of experimental steatohepatitis in mice. J Hepatol.

[20] Rahmani S (2016). Treatment of non-alcoholic fatty liver disease with curcumin: a randomized placebo-controlled trial. Phytother Res.

[21] Kocher A (2016). Highly bioavailable micellar curcuminoids accumulate in blood, are safe and do not reduce blood lipids and inflammation markers in moderately hyperlipidemic individuals. Mol Nutr Food Res.

[22] Parizadeh S.M., M. Ghayour-Mobarhan, ScientificWorldJournal.

[23] NHLBI Obesity Education Initiative Expert Panel on the Identification, Evaluation, and Treatment of Obesity in Adults (US). Clinical Guidelines on the Identification, Evaluation, and Treatment of Overweight and Obesity in Adults: The Evidence Report. Bethesda: National Heart, Lung, and Blood Institute; 1998 Sep. Available from: https://www.ncbi.nlm.nih.gov/books/NBK2003/.

[24] Malekzadeh R, Poustchi H (2011). Fibroscan for assessing liver fibrosis: an acceptable alternative for liver biopsy: Fibroscan: an acceptable alternative for liver biopsy. Hepat Mon.

[25] Ainsworth BE (2000). Compendium of physical activities: an update of activity codes and MET intensities. Med Sci Sports Exerc.

[26] Ghaffarpour M, Houshiar-Rad A, Kianfar H (1999). The manual for household measures, cooking yields factors and edible portion of foods.

[27] Kelany ME, Hakami TM, Omar AH (2016). Curcumin improves the metabolic syndrome in high-fructose-diet-fed rats: role of TNF-α, NF-κB, and oxidative stress. Can J Physiol Pharmacol.

[28] Maithilikarpagaselvi N (2016). Curcumin prevents inflammatory response, oxidative stress and insulin resistance in high fructose fed male Wistar rats: potential role of serine kinases. Chem Biol Interact.

[29] Ramirez-Tortosa MC (2009). Curcumin ameliorates rabbits's steatohepatitis via respiratory chain, oxidative stress, and TNF-α. Free Radic Biol Med.

[30] Cho J-W, Lee K-S, Kim C-W (2007). Curcumin attenuates the expression of IL-1β, IL-6, and TNF-α as well as cyclin E in TNF-α-treated HaCaT cells; NF-κB and MAPKs as potential upstream targets. Int J Mol Med.

[31] Shehzad A (2011). New mechanisms and the anti-inflammatory role of curcumin in obesity and obesity-related metabolic diseases. Eur J Nutr.

[32] Ziccardi P (2002). Reduction of inflammatory cytokine concentrations and improvement of endothelial functions in obese women after weight loss over one year. Circulation.

